# Analysis of Hemichannels and Gap Junctions: Application and Extension of the Passive Transmembrane Ion Transport Model

**DOI:** 10.3389/fncel.2021.596953

**Published:** 2021-04-07

**Authors:** Qiqian Wang, Shenquan Liu

**Affiliations:** School of Mathematics, South China University of Technology, Guangzhou, China

**Keywords:** electrical synapse, gap junction, hemichannel, connexin, innexin, docking, gating, pannexin

## Abstract

Electrical synaptic transmission is an essential form of interneuronal communication which is mediated by gap junctions that permit ion flow. Three gene families (connexins, innexins, and pannexins) have evolved to form gap junctional channels. Each gap junctional channel is formed by the docking of the hemichannel of one cell with the corresponding hemichannel of an adjacent cell. To date, there has been a lack of study models to describe this structure in detail. In this study, we demonstrate that numerical simulations suggest that the passive transmembrane ion transport model, based on the generality of ion channels, also applies to hemichannels in non-junctional plasma membranes. On this basis, we established a gap junctional channel model, which describes hemichannels' docking. We simulated homotypic and heterotypic gap junctions formed by connexins, innexins, and pannexins. Based on the numerical results and our theoretical model, we discussed the physiology of hemichannels and gap junctions, including ion blockage of hemichannels, voltage gating of gap junctions, and asymmetry and delay of electrical synaptic transmission, for which the numerical simulations are first comprehensively realized.

## 1. Introduction

Electrical synaptic transmission is an essential form of interneuronal communication mediated by *gap junctions*. Not only do gap junctions underlie the functional processes in the mammalian central nervous system, but they also play a crucial role in the physiology of poikilothermic vertebrates.

### 1.1. Gap Junctions

The evolution of multicellularity necessitated interactions to coordinate cell activity. Specialized and distinct structures emerged independently to provide direct communication between cells, in plants, by plasmodesmata, in fungi, by septal pores and by gap junctions in animals.

Gap junctions are clusters of intercellular channels where each channel results from the docking of the *hemichannel* of one cell with the *corresponding hemichannel* of an adjacent cell, thus allowing passive ion transport. Through gap junctional channels, action potentials can rapidly spread between excitable cells, such as cardiomyocytes and neurons. However, at certain stages of development, gap junctions exist in all tissues. Action potentials are generated by ion flow, and the transmission of electrical signals is essentially the passive transport of ions.

Gap junctional channels are regulated by several physiological agents, including transjunctional voltage, and intracellular and extracellular calcium ions (Bruzzone et al., 1996). The unique architecture of gap junctional channels is considered to be influenced by the electrical field. Homotypic gap junctional channels show symmetrical bell-shaped steady-state *G*_j_/*V*_j_ curves, which can be described by the Boltzmann relation (Harris et al., 1981). Based on a summary of experimental findings, the steady-state *G*_j_/*V*_j_ curves of heterotypic channels are generally asymmetric, with each hemichannel retaining roughly the properties of its homomeric combinations.

It is generally recognized that three gene families can form gap junctional channels: *connexins, innexins* and *pannexins*. In vertebrates, most gap junctions are formed by connexins. In invertebrates, innexin homologs were identified. Subsequently, they were identified in vertebrate genomes. In mammals and many vertebrates, pannexin genes expressed in the brain have also been identified.

### 1.2. Hemichannels

Although it was previously believed that unpaired hemichannels remained closed, evidence has suggested that hemichannels can be voltage gated by depolarization in non-junctional plasma membranes (Paul et al., 1991). Nowadays, it is known that most of the connexins can form functional hemichannels under proper conditions (Goodenough and Paul, 2003), and so can a series of innexins. However, although pannexins can form intercellular channels, this does not occur often. Therefore, whether pannexin channels are hemichannels is still controversial (Sosinsky et al., 2011).

#### 1.2.1. Connexins

Vertebrate gap junctional channels are mainly composed of connexins that oligomerize intracellularly into *connexons* in the membrane. Connexons in adjacent cells pair up to form intercellular channels, resulting in a 2–3 nm separation between the junctional membranes, which is negligible compared to the radius of the cell.

Over the evolutionary history, gap junctions formed by connexons have developed diverse functions. Several studies have demonstrated that some connexons in cellular membranes do not take part in gap junction formation, and evidence suggests that a subset of unpaired connexins can form open hemichannels with their own functions, with their presence in non-junctional cellular membranes being widely established (Goodenough and Paul, 2003).

#### 1.2.2. Innexins

Under low resolution electron microscopy, the morphologies of gap junction channels from native tissues of vertebrates and invertebrates appear analogous. However, it is now known that two genetically distant genes code gap junction proteins (Oshima et al., 2016). Direct cellular communication in vertebrates occurs through connexin-based gap junctions, whereas innexins form gap junctional channels in invertebrates (Raff et al., 2002). There is no significant sequence similarity between connexins and innexins, but some of their electrophysiological phenomena are similar. Whether connexins and innexins share a common ancestor or arose independently by convergent evolution remains to be determined (Oshima et al., 2016).

In addition to coordinating electrical activity, innexins' functions are diverse. For instance, Drosophila shak-B, an innexin gene, is known to additionally produce multiple products (Phelan, 2005).

#### 1.2.3. Pannexins

Highly unlike connexins, pannexins readily form single membrane channels, but does not often form intercellular channels. Although whether pannexin channels are hemichannels or not is still controversial, since they do form junction channels under some exceptional conditions (Sosinsky et al., 2011), we still include them in the scope of study.

Pannexins are expressed in almost all tissues and are especially abundantly in the central nervous system of vertebrates. There are three pannexin genes (Px1, Px2, and Px3) in mammals (Bruzzone et al., 2003). Pannexin sequence is moderately similar to the innexins but not to connexins. The way connexins and pannexins form structures within cells are also different from each other. For example, Cx46 forms hexameric gap junctions, while Px1 forms a heptameric hemichannel (Qu et al., 2020).

### 1.3. Docking Between Hemichannels

Hemichannels protrude approximately 20Å from the plasma membrane. Two hemichannels dock end-to-end to form a junctional channel. The binding reaction must be initiated by contact between hemichannels. This contact must be stable and sealed, exclude ions, and allow pore-opening.

The stability of the junctional channel structure suggests a tight interlocking arrangement between two paired hemichannels (Harris, 2001). That is, the docking ends of paired hemichannels should be relatively fixed; therefore, state change of one must affect the other. Polarity reversal experiments also suggested that the gates on each side of a junctional channel were not independent, but were rather influenced by each other.

### 1.4. Signal Transmission

Gap junctional channels support direct communication between cells and are an integral part of signal transmission. For instance, all retinal cells communicate via gap junctions (Hornstein et al., 2005). However, it is not well-known how the hemichannels function to generate signal transmission and how the structure of gap junctions impacts the characteristics of signal transmission.

As mentioned above, each intercellular channel forms a pathway for direct ion transfer, thus generating an ion flow between cells. Although previously viewed as symmetrical channels, research on invertebrates has revealed that unique gap junction-forming proteins may asymmetrically contribute from each side of the synapse. Some findings suggest that this asymmetry could also exist in the vertebrate nervous systems (Miller et al., 2017). This structural asymmetry generates functional asymmetry in ion flow through gap junctions (Phelan et al., 2009).

## 2. Foundation

### 2.1. Operators

In this paper, we use several operation symbols for sequences and matrices to simplify expressions: (*a*_*i,j*_) ◦ (*b*_*i,j*_) = (*a*_*i,j*_ · *b*_*i,j*_), (*a*_*i*_) ◦ (*b*_*i,j*_) = (*b*_*i,j*_) ◦ (*a*_*i*_) = (*a*_*i*_ · *b*_*i,j*_), (*a*_*i*_) ◦ (*b*_*i*_) = (*a*_*i*_ · *b*_*i*_); ${\left(a_{i}\right)}^{\circ \left(b_{i}\right)}=\left(a_{i}^{b_{i}}\right)$, ${\left(a_{i}\right)}^{\circ b}=\left(a_{i}^{b}\right)$; (*a*_*i,j*_) ⊘ (*b*_*i,j*_) = (*a*_*i,j*_/*b*_*i,j*_), (*a*_*i*_) ⊘ (*b*_*i*_) = (*a*_*i*_/*b*_*i*_); $\sum \left(a_{i}\right)=\underset{i}{\sum }a_{i}$, $\sum \left(a_{i,j}\right)=\left(\underset{i}{\sum }a_{i,j}\right)$, (*a*_*i*_)[*k*] = *a*_*k*_.

### 2.2. Model of Passive Transmembrane Ion Transport

Here, we briefly introduce the key elements of the passive transmembrane ion transport model. This model is based on the generality of ion channels and is applicable for various ion channels in different cells, such as plant cells, myocytes, and neurocytes. A more detailed discussion, derivation, and numerical experiments can be found in our previous paper (Wang and Liu, 2019).

As generally understood, an ion channel can be abstracted as a combination of filter and gate,

(1) $$\begin{array}{l} \rho =\text{f}\circ \text{g}\circ \varrho , \end{array}$$

where ***ρ*** = (ρ_*h,i*_), ρ_*h,i*_ is the selective permeability of channel *h* for ion *i*; ***f*** = (*f*_*h,i*_), *f*_*h,i*_ describes the filter of channel *h* to ion *i*; ***g*** = (*g*_*h*_), *g*_*h*_ = *v*_*h*_*w*_*h*_ describes the gate of channel *h*, and ***ϱ*** = (ϱ_*h,i*_), ϱ_*h,i*_ is the standard selective permeability of channel *h* to ion *i*. Therefore, channel states can be described by ***f***, ***v***, and ***w***. When all external conditions remain constant, the states of channels tend to be stable,

$$\begin{array}{l} \underset{t\to \infty }{\lim}\left(\begin{array}{c} \text{v}\\ \text{w}\\ \text{f} \end{array}\right)=\left(\begin{array}{c} \bar{\text{v}}\\ \bar{\text{w}}\\ \bar{\text{f}} \end{array}\right). \end{array}$$

The stable states depend on the ionic flux (indicated by **ϕ**) and internal ionic density (indicated by **ϑ**) detected by the channels,

(2) $$\begin{array}{l} \left(\begin{array}{c} \bar{\text{v}}\\ \bar{\text{w}}\\ \bar{\text{f}} \end{array}\right)=H\left(\begin{array}{c} \vartheta \\ \phi \end{array}\right)\equiv \left[\begin{array}{c} 1-{\left(\text{cos}\vartheta \right)}^{\circ \eta }\\ \pi^{-1}\text{acot}\phi ⊘\vartheta \\ exp\left(-\kappa \circ \vartheta^{\circ 2}\right) \end{array}\right]. \end{array}$$

where **ϕ** = (ϕ_*h*_), and ϕ_*h*_ points out the influence of detected ionic flux on density of channel *h*; **ϑ** = (ϑ_*h*_), ϑ_*h*_ points out the difference between detected ionic density and optimal density of channel *h*; **η** = (η_*h*_), η_*h*_ ∈ (0, ∞) is the tolerance of channel *h* to ionic density deviation, and describes the degree of which the channel allows the ion density to deviate from the optimal value; and **κ** = (κ_*h,i*_), κ_*h,i*_ is the inactivation coefficient of channel *h* occupied by ion *i*.

When we say *h* ∈ **H**, we mean *h* belongs to **H** channels. For example, when we say *h*_1_ ∈ Px1 and *h*_2_ ∈ Px1, we mean *h*_1_ and *h*_2_ are both Px1 channels. Obviously, Px1 ∪ Px2 ∪ Px3 ⊂ Px.

### 2.3. Fundamental Assumptions of Gap Junctions

Summarizing the previously presented information on gap junctions and hemichannels:

- Functional hemichannels in the non-junctional plasma membrane have their own functions.
- Each gap junctional channel is formed by the docking of a hemichannel in one cell with the corresponding hemichannel in an adjacent cell.
- The docking is stable and sealed, excludes ions and allows pore opening.
- Evidence exists for a dissociation between docking and opening. The gate of the hemichannel is supposed to lay at the intracellular end.
- The docking ends of paired hemichannels are relatively fixed; therefore, state change of one must affect the other.
- Hemichannels protrude approximately 20Å from the plasma membrane.
- There is a 2–3 nm separation between the junctional membranes.

Based on the above information, we made the following reasonable assumptions:

** Assumption 1**. Hemichannels conform to the basic assumptions of the passive transmembrane ion transport model.

** Assumption 2**. The docking is sealed such that ions do not leak from the junctions.

** Assumption 3**. The state of two paired hemichannels affect each other.

** Assumption 4**. The gap excludes external ions, and the gap distance is negligible.

According to Assumption 1, hemichannels can be described by the model of passive transmembrane ion transport. We now proceed to describe the model of the docking of paired hemichannels.

## 3. Model of Gap Junctional Channels

As presented above, establishing a gap junctional channel model means establishing a model for the docking of two hemichannels. As such, we marked the corresponding hemichannel of *h* as *h*_¶_. For convenience of presentation, we set ¶(*x*_*h*_) = (*x*_*h*_¶__). As the two cells are tightly connected, their internal charges also interact.

We mark the junctional channel formed by the docking of hemichannels *h*_1_ and *h*_2_ as (*h*_1_, *h*_2_). For example, the docking of *h*_1_ (∈ Cx43) and *h*_2_ (∈ Cx46) forms (*h*_1_, *h*_2_), which is a Cx43/Cx46 gap junctional channel. In strict mathematical language, it should be written as Cx43 × Cx46, but in this paper we will still follow the physiological idiom.

### 3.1. Electric Field

We previously discussed the ideal electric field distribution of a single cell (Wang and Liu, 2019). We now calculate the electric field at a gap junction. The ionic density on both sides of the gap junction will then be deduced. According to Assumption 4, at a gap junction, the electric field arises from the electrons in the junctional cells, and the electric field between the junctional membranes (connected by hemichannels *h* and *h*_¶_) can be considered as parallel,

(3) $$\begin{array}{l} E\left(\begin{array}{c} {\text{c}}_{h_{¶}}\\ {\text{c}}_{h} \end{array}\right)\equiv \frac{1}{2\varepsilon }F\text{z}Q\left({\text{c}}_{h}-{\text{c}}_{h_{¶}}\right), \end{array}$$

where the direction goes from *h* to *h*_¶_, **c** = (*c*_*i*_), *c*_*i*_ is the concentration of ion *i*, and

(4) $$\begin{array}{l} Q\text{c}\equiv r^{-2}\int_{0}^{r}\text{c}\cdot x^{2}\text{d}x \end{array}$$

is the total amount of intracellular ions, where *r* is the cell radius and *x* is the distance to the center of the cell. It is obvious that E(*c*_*h*_¶__; *c*_*h*_) + E(*c*_*h*_; *c*_*h*_¶__) = 0. At the gap junction, the external ionic density does not need to be calculated, while the internal ionic density of *h* is approximately

(5) $$\begin{array}{l} S_{\text{in}}\left(\begin{array}{c} {\text{c}}_{h_{¶}}\\ {\text{c}}_{h} \end{array}\right)\equiv \lambda \circ A_{\text{in}}{\text{c}}_{h}\circ \left[1-E\left(\begin{array}{c} {\text{c}}_{h_{¶}}\\ {\text{c}}_{h} \end{array}\right)\cdot \lambda \circ \frac{\text{z}F}{2RT}\right]⊘\left[1+E\left(\begin{array}{c} {\text{c}}_{h_{¶}}\\ {\text{c}}_{h} \end{array}\right)\cdot \lambda \circ \frac{\text{z}F}{2RT}\right], \end{array}$$

where

(6) $$\begin{array}{l} A_{\text{in}}\text{c}\equiv 3r^{-3}\int_{0}^{r}\text{c}\cdot x^{2}\text{d}x, \end{array}$$

is the average internal ion concentration (distribution).

### 3.2. Permeability

For a channel *h*, in general, we can obtain the average permeability **ρ**_*h*_ on the membrane, where the surface area is *S*_*h*_. The total permeability of the entire cell is

(7) $$\begin{array}{l} P_{h}=\rho_{h}S_{h}. \end{array}$$

The docking of the two hemichannels is not a simple series connection, instead, the real situation is complex. We considered the unpaired hemichannel *h* in the open state as a cylinder (radius of the cross section *r*_*h*_, height *l*_*h*_); After pairing, the two hemichannels (*h,h*_¶_) are connected to form a circular truncated cone (height *l*_*h*_ + *l*_*h*_¶__, with cross section radiuses *r*_*h*_ and *r*_*h*_¶__). It is easy to derive the axial conductivity for the circular truncated cone,

(8) $$\begin{array}{l} P_{h,h_{¶}}=D\cdot \frac{\pi r_{h}r_{h_{¶}}}{l_{h}+l_{h_{¶}}}. \end{array}$$

While the conductivity of a cylinder (unpaired hemichannel) is

(9) $$\begin{array}{l} P_{h}=D\cdot \frac{\pi r_{h}^{2}}{l_{h}}. \end{array}$$

If *l*_*h*_ = *l*_*h*_¶__ = *l*, then

(10) $$\begin{array}{l} P_{h,h_{¶}}=\frac{{\left(P_{h}\circ P_{h_{¶}}\right)}^{\circ \frac{1}{2}}}{2}. \end{array}$$

According to Assumption 2, the ion flow from *h* to *h*_¶_ would be,

(11) $$\begin{array}{l} J_{h,h_{¶}}=P_{h,h_{¶}}\circ \left[S_{\text{in}}\left(\begin{array}{c} {\text{c}}_{h_{¶}}\\ {\text{c}}_{h} \end{array}\right)-S_{\text{in}}\left(\begin{array}{c} {\text{c}}_{h}\\ {\text{c}}_{h_{¶}} \end{array}\right)\right]. \end{array}$$

### 3.3. Gating

According to Assumption 3, the paired hemichannels *h* and *h*_¶_ are not independent of each other. The state of one affects the other. In the model of passive transmembrane ion transport, the state of channel *h* depends on ϑ_*h*_. When ϑ_*h*_ = 0, the channel tends to be at rest. Assuming that the torsion of *h*_¶_ by *h* is 0 when hemichannel *h* remains or tends to be at rest, as ϑ_*h*_ = 0, then a simple relationship would be

(12) $$\begin{array}{l} \left(\begin{array}{c} \bar{\text{v}}\\ \bar{\text{w}}\\ \bar{\text{f}} \end{array}\right)=H\left[\begin{array}{c} \vartheta +¶\left(\text{u}\circ \vartheta \right)\\ \phi \end{array}\right]. \end{array}$$

Here, *u*_*h*_ (≥ 0) describes the effect of *h* on *h*_¶_ in junctional channel (*h, h*_¶_). When *u*_*h*_ = 0, *h* has no effect on *h*_¶_; the larger *u*_*h*_ is, the greater the effect of *h* on *h*_¶_; the smaller *u*_*h*_ is, the more the independence of *h*_¶_. If *u*_*h*_ is related to ϑ_*h*_, the greater the value of |ϑ_*h*_|, the more significant the effect of *h* on *h*_¶_. Thereafter,

(13) $$\begin{array}{l} u_{h}=\varpi_{h}|\vartheta_{h}{|}^{n_{h}} \end{array}$$

is a natural consequence, where the parameters (ϖ_*h*_, *n*_*h*_) depend on the connection of (*h, h*_¶_), and set *u*_*h*_ = 0 (or ϖ_*h*_ = 0) if *h* is not paired. If *u*_*h*_ = *u*_*h*_¶__ = 0, *h* and *h*_¶_ are completely independent. In particular, if *h* and *h*_¶_ are symmetrical and the internal ion concentrations of the two cells are equal, then for any *V*_j_, there is ϑ_*h*_ = −ϑ_*h*_¶__. In this situation, if *u*_*h*_ = *u*_*h*_¶__ = 1, then ϑ_*h*_ + *u*_*h*_¶__ϑ_*h*_¶__ = 0 and ϑ_*h*_¶__ + *u*_*h*_ϑ_*h*_ = 0, according to Equation (12), both hemichannels *h* and *h*_¶_ will lose their sensitivity completely. Hence, for a docking of *h* and *h*_¶_, there should be *u*_*h*_, *u*_*h*_¶__ ∈ (0, 1).

### 3.4. Voltage and Current

Considering a cell, we can estimate its membrane potential based on the intracellular ion concentrations,

(14) $$\begin{array}{l} V_{\text{m}}=V\text{c}\equiv \frac{F\delta_{\text{m}}}{\varepsilon_{\text{m}}}\text{z}Q\text{c}, \end{array}$$

where δ_m_ is the membrane thickness. Given a stimulation voltage *V*_s_, the stimulation current would be

(15) $$\begin{array}{l} I_{\text{s}}=\frac{V_{\text{s}}-V_{\text{m}}}{R_{\text{s}}}, \end{array}$$

where *R*_s_ is the electrode resistance. The detailed conversion methods of current and ionic flux are available in our previous study (Wang and Liu, 2019).

For a gap junction between two given cells L and R, a double voltage-clamp procedure is commonly used to measure junctional current (Spray et al., 1981). Each of the two cells is independently voltage-clamped. Typically, voltage steps _*V*_s_(L)_ are delivered to cell L, while cell R is held at a constant potential, _*V*_s_(R)_. Junctional voltage is the difference between the membrane potentials of cells L and R,

(16) $$\begin{array}{l} V_{\text{j}}={V_{\text{m}}}_{\left(L\right)}-{V_{\text{m}}}_{\left(R\right)}. \end{array}$$

Further, there is a potential difference between the two voltage-clamps, denoted as

(17) $$\begin{array}{l} \Delta V_{\text{s}}={V_{\text{s}}}_{\left(\text{L}\right)}-{V_{\text{s}}}_{\left(\text{R}\right)}. \end{array}$$

*V*_j_ always lags behind Δ*V*_s_, and *V*_j_ → Δ*V*_s_. Given a junctional channel, the junctional current can be calculated by junctional ionic flow,

(18) $$\begin{array}{l} I_{\text{j}}=F\text{z}J. \end{array}$$

Consequently, the conductance of this junctional current can be calculated by

(19) $$\begin{array}{l} G_{\text{j}}=\frac{I_{\text{j}}}{V_{\text{j}}}. \end{array}$$

This conductance is a virtual concept, a variable describing the state change in the junctional channel since a real conductor between the two cells does not actually exist.

## 4. Results and Discussion

### 4.1. Parameters

#### 4.1.1. Solution

The solution of numerical experiments in this study (in mmol/L): [I-1] NaCl 2, KCl 92, Mg(OH)_2_ 0.5, Ca-EGTA 1; [I-2] NaCl 1, KCl 90, Ca(NO)_3_ 0.75; [I-3] NaCl 2, KCl 92, Mg(OH)_2_ 0.5, Ca-EGTA 2; [I-4] NaCl 9, NaOH 1, KCl 120, CsCl 1, Ca-EGTA 1, MgCl_2_ 2; [E-1] NaCl 139, NaOH 1, KCl 5, CsCl 1, Ca-EGTA 1, MgCl_2_ 1; [ND96] NaCl 96, KCl 2, Mg(OH)_2_ 1, Ca-EGTA 1.8; [ND96-1] NaCl 96, KCl 2, Ca-EGTA 0.5; [ND96-2] NaCl 96, KCl 2, Ca-EGTA 5; [Barth's] NaCl 88, KCl 1, NaHCO_3_ 2.4, Ca(NO_3_)_2_ 0.33, CaCl_2_ 0.41, MgSO_4_ 0.82.

#### 4.1.2. Ion Parameters

The ionic radiuses (pm) used in the numerical simulations: Na^+^ 102, Mg^2+^ 72, Cl^−^ 181, K^+^ 138, Ca^2+^ 100, Cs^+^ 167, OH^−^ 137.

#### 4.1.3. Cell Parameters

Temperature = 310 K, membrane thickness = 6 nm, relative membrane permittivity = 7, radius = 50 μm.

#### 4.1.4. Channel Parameters

All the parameters of the passive transmembrane ion transport model are recorded in Tables 1–4, of which the definitions can be found in our previous paper (Wang and Liu, 2019). The parameters of the gap junction model are recorded in Table 5. The parameters unassigned defaults to 0.

**Table 1:** Filter state parameters.

***h***	$\gamma_{h}\left({\text{s}}^{-1}\right)$	**κ_*h,i*_**				
		**Na^+^**	**K^+^**	**Cs^+^**	**Ca^2+^**	**Cl^−^**
Cx32	–	–	–	–	–	–
Cx36	–	–	–	–	–	–
Cx43	–	–	–	–	–	–
Cx46	–	–	–	–	–	–
ShakBL	–	–	–	–	–	–
ShakBN16	–	–	–	–	–	–
Px1	2.0	12	16	–	–	16

**Table 2:** Standard selective permeability $\varrho_{h,i}\left({\text{ms}}^{-1}\right)$ of filter.

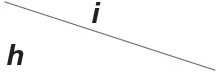	**Na^+^**	**K^+^**	**Ca^2+^**	**Mg^2+^**	**Cl^−^**
Cx32	0.50	0.40	0.40	0.40	0.10
Cx36	0.52	0.40	0.40	0.40	0.10
Cx43	0.15	0.10	0.10	0.10	0.01
Cx46	0.52	0.40	0.40	0.40	0.10
ShakBL	0.52	0.40	0.40	0.40	0.10
ShakBN16	0.52	0.40	0.40	0.40	0.10
Px1	0.45	0.40	0.40	0.40	0.10
Na_v_	100	10	–	–	–
K_v_	–	20	–	–	–
Cl_v_	–	–	–	–	10

**Table 3:** Properties of gate and sensor.

**h**	$\alpha_{h}\left({\text{s}}^{-1}\right)$	$\beta_{h}/\left(1+\delta_{h}\right)\left({\text{s}}^{-1}\right)$	**δ_*h*_**	${\bar{\sigma }}_{h}\left(\text{nmol}/{\text{m}}^{2}\right)$	**τ_*h*_/(ϱ_*h*_·s_*h*_)**	**η_*h*_**
Cx32	0.5	2.0	10	0.120	50	3.0
Cx36	1.0	3.0	100	0.130	50	3.0
Cx43	2.0	3.0 × 10^3^	1.0 × 10^−3^	0.135	100	3.0
Cx46	1.2	5.0	2.0	0.135	50	3.0
ShakBL	6.0	6.0 × 10^3^	1.0 × 10^−2^	0.135	50	3.0
ShakBN16	6.0	6.0 × 10^3^	1.0 × 10^−2^	0.135	50	3.0
Px1	20	20	100	0.36	50	3.0
Na_v_	5.0 × 10^3^	5.0 × 10^4^	5.0 × 10^−3^	1.9	1.0 × 10^6^	5.0
K_v_	5.0 × 10^2^	5.0 × 10^4^	5.0 × 10^−3^	30	1.0 × 10^6^	2.0
Cl_v_	1.0 × 10^3^	5.0 × 10^4^	5.0 × 10^−3^	56	1.0 × 10^6^	2.0

The sensitivity and permeability parameters of inductor are shown in Tables 2, 4

**Table 4:** Sensitivity parameters s_*h,i*_.

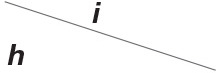	**Na^+^**	**K^+^**	**Ca^2+^**	**Mg^2+^**	**Cl^−^**
Cx32	–	–	0.3	1.0	–
Cx36	–	–	1.0	–	–
Cx43	–	–	1.0	1.0	–
Cx46	–	–	1.0	–	–
ShakBL	–	–	1.0	–	–
ShakN16	–	–	1.0	–	–
Px1	–	–	1.0	–	–
Na_v_	1.0	–	–	–	–
K_v_	–	1.0	–	–	–
Cl_v_	–	–	–	–	1.0

**Table 5:** Junction parameters.

**Junction (*h*; *h*_**¶**_)**	**ϖ_*h*_**	***n*_*h*_**	**ϖ_*h*¶_**	***n*_*h*¶_**
Cx32/Cx32	19:	3:0	19:	3:0
Cx36/Cx36	3:0	2:0	3:0	2:0
Cx43/Cx43	4:0	2:0	4:0	2:0
Cx46/Cx46	13:	3:0	13:	3:0
Cx43/Cx46	4:0	2:0	13:	3:0
ShakBL/ShakBL	4:2	2:0	4:2	2:0
ShakBN16/ShakBN16	1:5	0:5	1:5	0:5
ShakBL/ShakBN16	4:2	2:0	1:5	0:5
Px1/Px1	0:9	0:0	0:9	0:0

### 4.2. Unpaired Hemichannels

External calcium ions can induce reversible conformational changes in hemichannel structure and affect the voltage sensitivity of gating, as the activation of hemichannels depends on ${\left[\text{C}{\text{a}}^{2+}\right]}_{\text{ex}}$ (Gómez-Hernàndez et al., 2003). For example, extracellular calcium ion can block Cx32 hemichannels.

#### 4.2.1. Blockage and Sensitivity

With an appropriate concentration of external solution, human Cx32 hemichannels expressed in *Xenopus* oocytes can be opened by raising the membrane potential. Additionally, variations in ${\left[\text{C}{\text{a}}^{2+}\right]}_{\text{ex}}$ regulate Cx32 hemichannels. In normal ND96 solution (${\left[\text{C}{\text{a}}^{2+}\right]}_{\text{ex}}=1.8\text{mM}$, ${\left[\text{M}{\text{g}}^{2+}\right]}_{\text{ex}}=1\text{mM}$), depolarizing pulses slowly activate outward currents, which increase with the degree of depolarization. With a return to the holding potential (*V*_m_ = −40 mV), the currents turn inward and the hemichannels slowly close. When ${\left[\text{C}{\text{a}}^{2+}\right]}_{\text{ex}}=5\text{mM}$, and ${\left[\text{M}{\text{g}}^{2+}\right]}_{\text{ex}}=0\text{mM}$, the amplitudes of the hemichannel currents are maintained at a similar level. Substitution of external Ca^2+^ with Mg^2+^ also inhibits Cx32 hemichannel activation. It is worth noting that lowering ${\left[\text{C}{\text{a}}^{2+}\right]}_{\text{ex}}$ to 0.5 mM increases the amplitudes of depolarization-induced currents, more than two-fold. This increase is a sudden and large rise rather than a smooth change (Gómez-Hernàndez et al., 2003).

**Figure 1:**
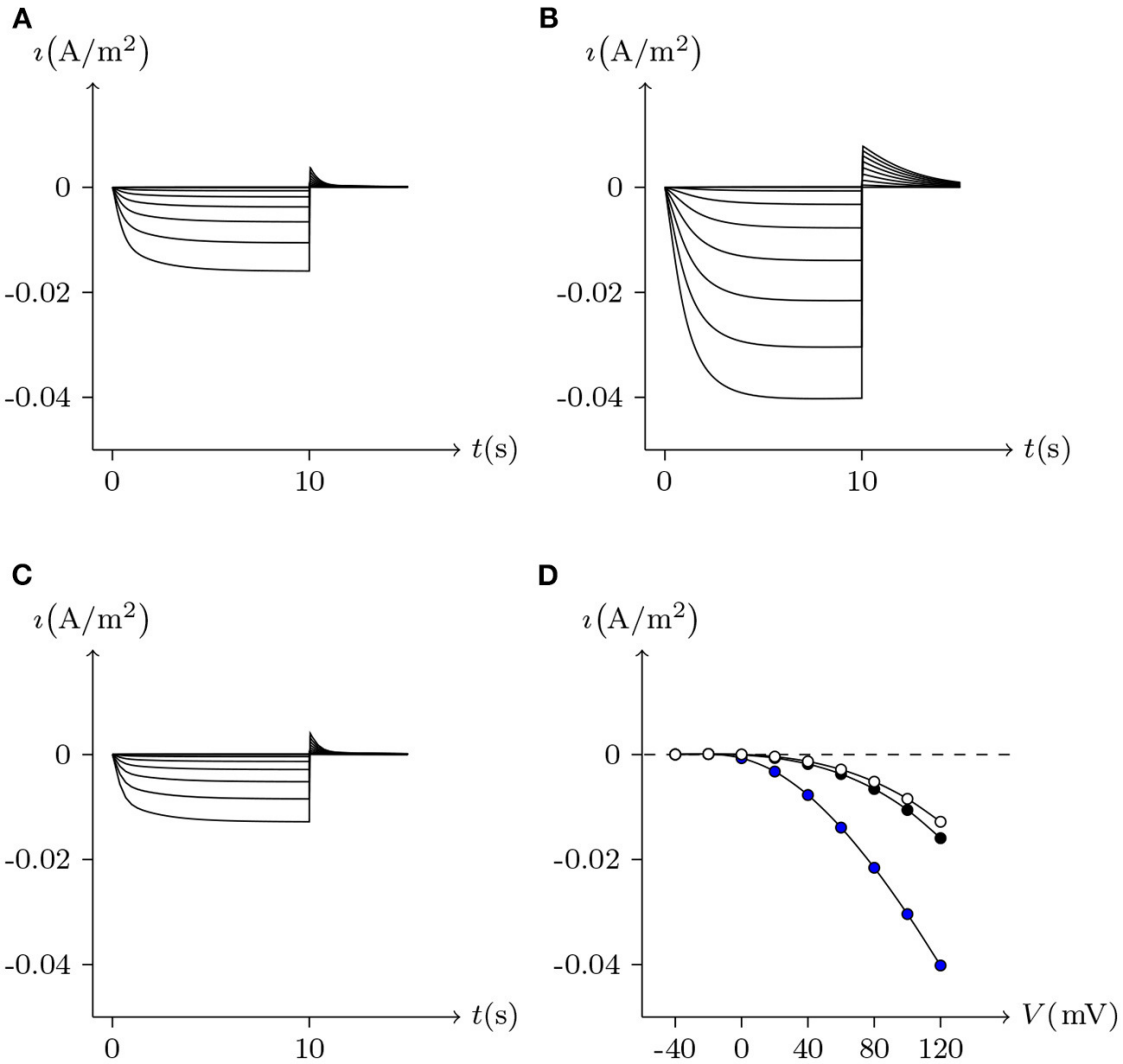
Simulated activating currents recorded from an isolated cell expressing Cx32 in normal, low and high ${\left[\text{C}{\text{a}}^{2+}\right]}_{\text{ex}}$ bath solutions. Unpaired hemichannel: Cx32. Internal solution: I-1. Patch clamp: *R*_s_ = 0.32 MΩ; *V*_s_ = −40 mV for *t*/s ∉ [0, 10), *V*_s_ = −40 mV to 120 mV for *t*/s ∈ [0, 10). **(A)** External solution: ND96 ([Ca^2+^] = 1.8 mM, [Mg^2+^] = 1.0 mM). **(B)** External solution: ND96-1 ([Ca^2+^] = 0.5 mM). **(C)** External solution: ND96-2 ([Ca^2+^] = 5.0 mM). **(D)** The points are recorded at *V*_s_ = *V* and *t* = 10 s, black for **(A)**, blue for **(B)**, and white for **(C)**.

The above phenomena observed in electrophysiological experiments suggest that the external Ca^2+^ blocks voltage gating of Cx32 hemichannels, which can be effectively simulated with the passive transmembrane ion transport model (see Figure 1), where the parameters (*w*_Cx32_ ≈ 0) indicate that it is actually the calcium and magnesium influxes that close the gate.

Based on the simulation results, it can be speculated that when the intracellular calcium ion concentration is extremely low, extracellular calcium ion blockage will disappear, a situation observed in Cx43-expressing glioma cells. Hemichannel responses were triggered at ${\left[\text{C}{\text{a}}^{2+}\right]}_{\text{in}}<500\text{nM}$, and an elevation of ${\left[\text{C}{\text{a}}^{2+}\right]}_{\text{in}}$ triggered hemichannel opening. These responses disappeared with larger ${\left[\text{C}{\text{a}}^{2+}\right]}_{\text{in}}$ transients (Vuyst et al., 2009).

#### 4.2.2. Activation

**Figure 2:**
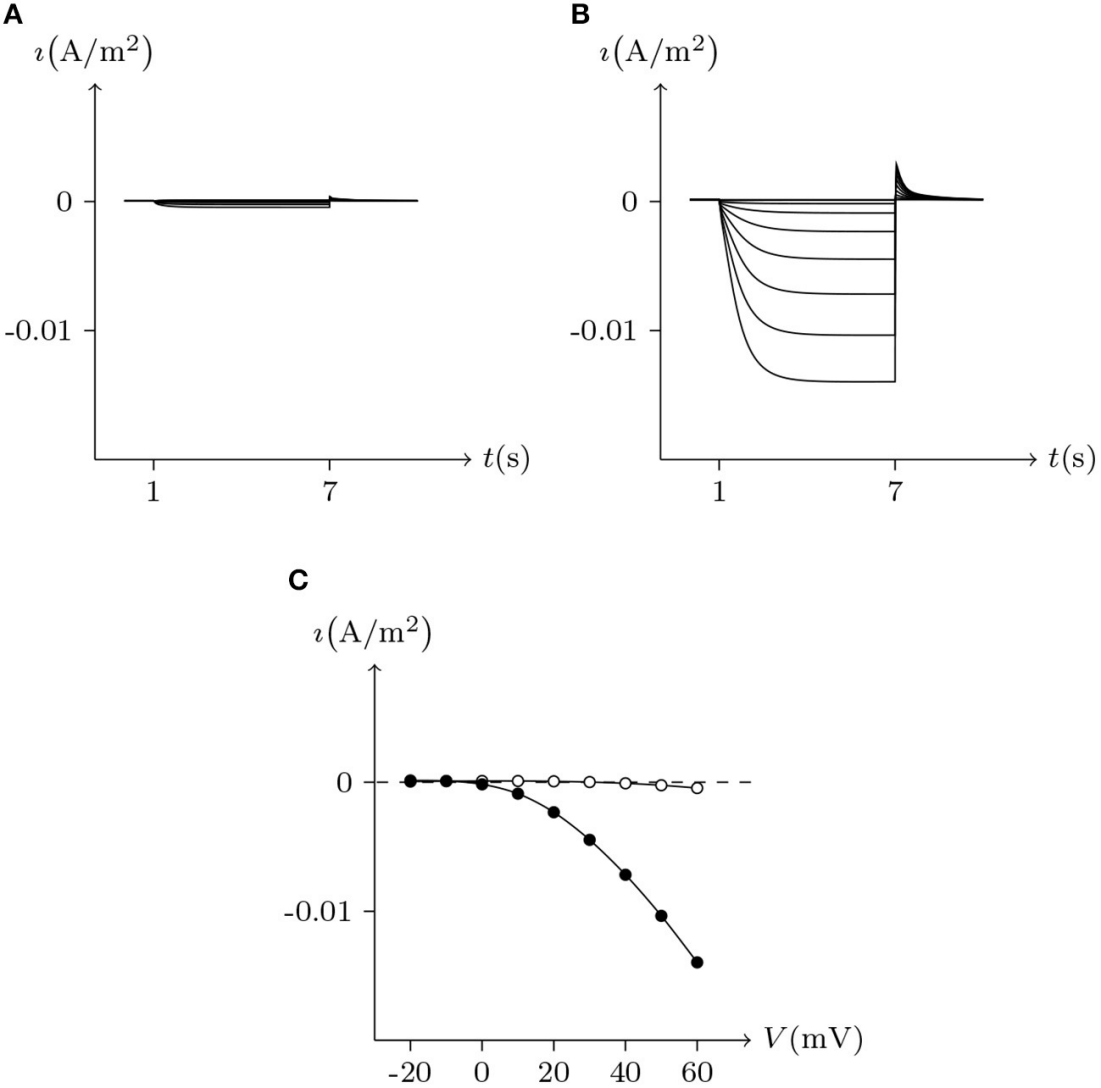
Numerical simulation of membrane currents recorded from individual cells expressing Cx43 and Cx46 individually, in response to depolarizing voltage steps from a holding potential of −20 mV, and stepped in 10 mV increments from −20 mV to +60 mV. Internal solution: I-2. External solution: Barth's. Patch clamp: *R*_s_ = 1.0 MΩ; *V*_s_ = −20 mV for *t*/s ∉ [1, 7], *V*_s_ = −20 mV to 60 mV for *t*/s ∈ [1, 7]. **(A)** Unpaired hemichannel: Cx43. **(B)** Unpaired hemichannel: Cx46. **(C)** Current-voltage relationships for cells individually expressing Cx43 and Cx46. The points are recorded at *t* = 7 s, white for **(A)** (Cx43) and black for **(B)** (Cx46). Compared to those for Cx46, the outward currents typical of hemichannel activity were absent from oocytes expressing Cx43.

Figure 2 simulates the experiments of membrane currents from individual Xenopus oocytes expressing Cx43 and Cx46. Oocytes expressing Cx43 did not exhibit significant hemichannel activity. This is consistent with previous observations suggesting that Cx43 hemichannels have a very low opening probability in Barth's solution (Vuyst et al., 2009). In contrast, depolarizing pulses significantly activate outward currents of cells expressing Cx46 (Quan et al., 2010).

In contrast to most connexin hemichannels, Px1 hemichannels are always active at physiological ${\left[\text{C}{\text{a}}^{2+}\right]}_{\text{ex}}$, the reason for which can be found in model parameters. Take Cx32 as an example to compare with Px1. For ${\bar{\sigma }}_{\text{Px}1}\approx 3{\bar{\sigma }}_{\text{Cx}32}$ (see Table 3, where ${\bar{\sigma }}_{h}$ is the optimum density of *h*), Px1 channels tend to maintain an internal calcium ion density of about 3 times of that of Cx32 (magnesium-free). Hence at physiological ${\left[\text{C}{\text{a}}^{2+}\right]}_{\text{ex}}$, Px1 would not be blocked by calcium ion influx when *V*_s_ > *V*_∞_.

**Figure 3:**
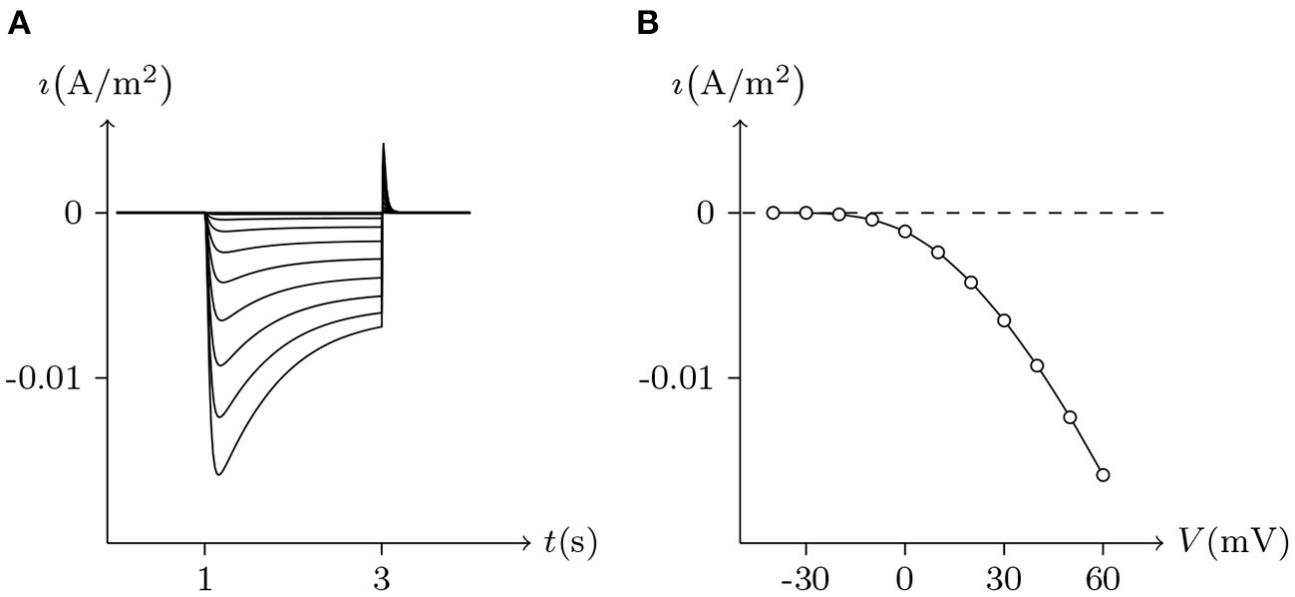
Simulated whole-cell membrane currents recorded from single cell expressing Px1. Unpaired hemichannel: Px1. External solution: ND96. Internal solution: I-3. Patch clamp: *R*_s_ = 32 kΩ; *V*_s_ = −40 mV for *t*/s ∉ [1, 3], *V*_s_ = −40 to 60 mV for *t*/s ∈ [1, 3]. **(A)** Activating outward currents evoked by depolarizing pulses. **(B)** Current-voltage relationship where points are recorded at the peaks for *t*/s ∈ [1, 3].

Further, the activation current curves of Px1 hemichannels are considerably different to those of the connexin family: Px1 hemichannels activate rapidly, and the ionic flux reaches its maximum in a distinctly short time. Outward currents are significant and consistent when *V*_s_ > −20 mV, reaching a peak within (30, 60)ms and then slightly and slowly declining. This rectification became more pronounced with increasing of *V*_s_ (Bruzzone et al., 2003). Figure 3 simulates these phenomena using the model of passive transmembrane ion transport. Filter parameters **γ** and **κ** (see Table 1) indicate that the function of unpaired Px1 channels on ion transport is different from that of Cx32, Cx43, and Cx46. Decline in the ion selectivity of Px1 after activation is significant. In contrast, the ion selectivity of other hemichannels in this paper is essentially constant.

The above numerical simulations verified the applicability of the model of passive transmembrane ion transport to unpaired hemichannels.

### 4.3. Gap Junctions

The permeabilities of gap junction channels are governed by hemichannels. The dependence of *G*_j_ (junctional conductance) on *V*_j_ (junctional voltage) has been extensively estimated. The conductance of all junctions is *V*_j_ sensitive because *V*_j_ is highly correlated with the ion distribution on both sides of the gap junctional channels. The model of gap junctional channels in this study can be used to explore the functional properties of gap junctions formed by various hemichannels combinations.

#### 4.3.1. Formed by Connexins

**Figure 4:**
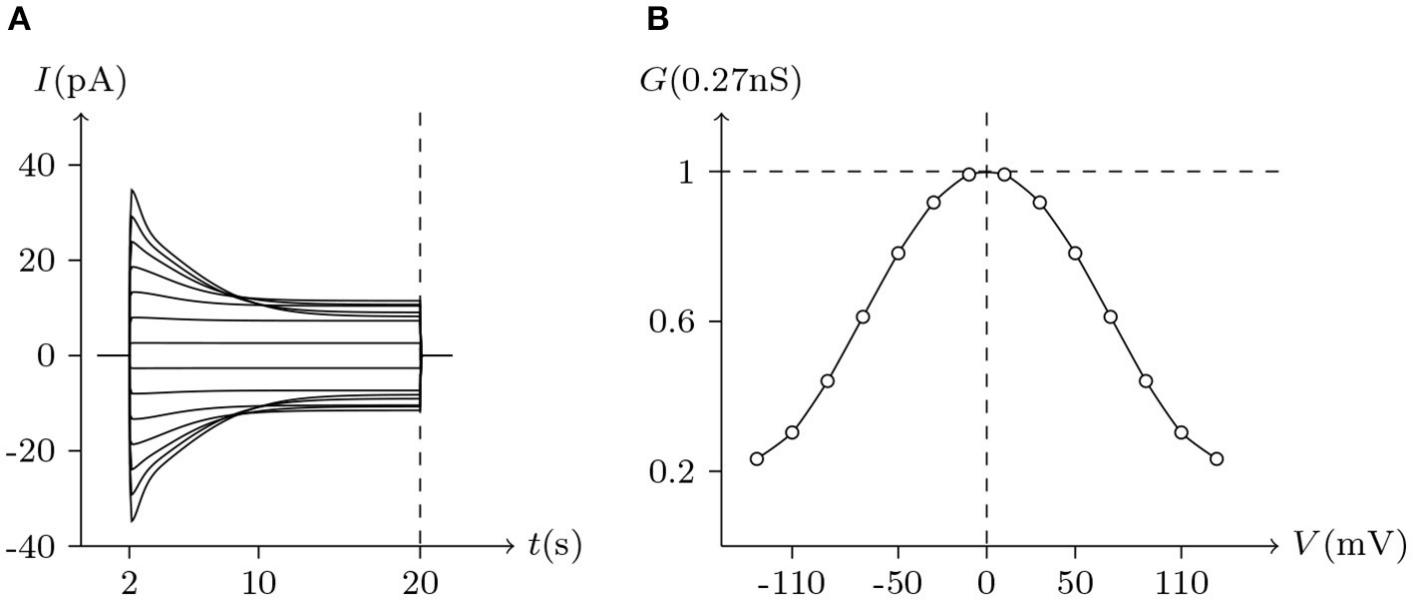
Simulated *V*_j_ gating property of Cx32/Cx32 gap junctional channels. Gap junctional channel: Cx32/Cx32. Internal and external solution: equal as in Figure 1. Patch clamps: *R*_s(L)_ = *R*_s(R)_ = 0.3 MΩ; Δ*V*_s_ = 0 mV for *t*/s ∉ [2, 20], Δ*V*_s_ = −130 to +130 mV for *t*/s ∈ [2, 20]. **(A)** Junctional currents induced by *V*_j_ pulses. **(B)** Graph of the steady-state (*V*_j_, *G*_j_) relationship where points are recorded at *t* = 20 s.

**Figure 5:**
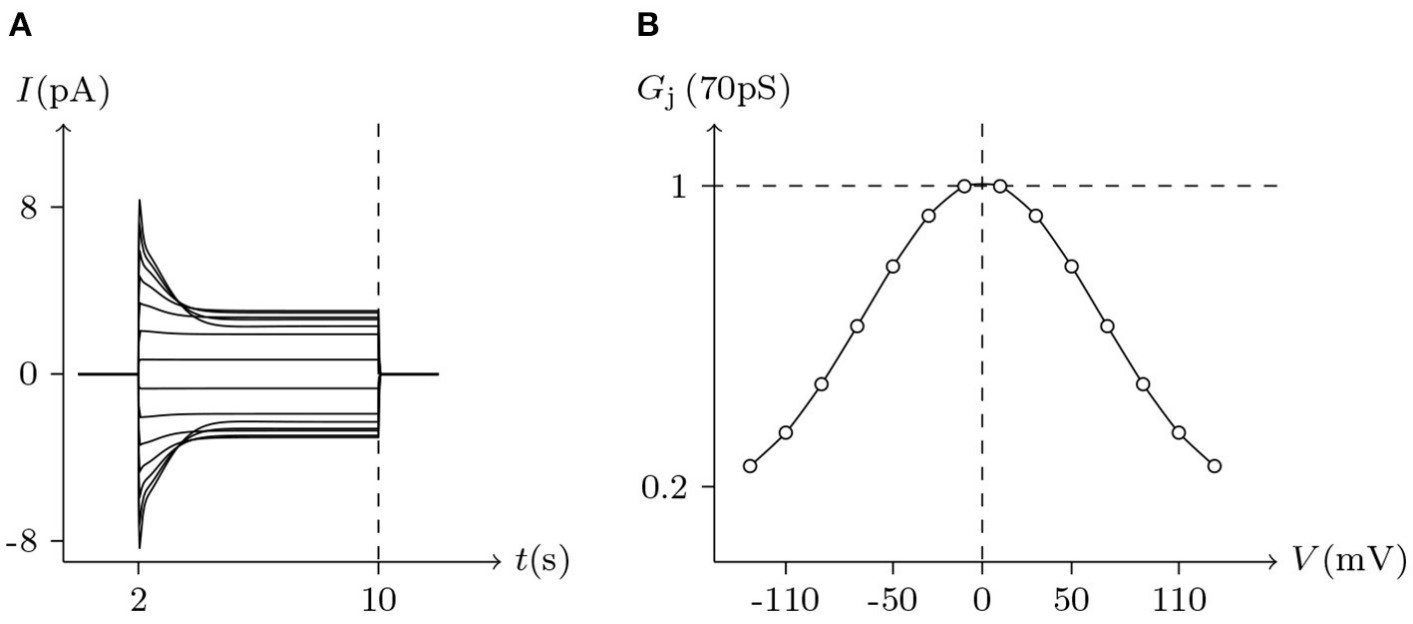
Simulated *V*_j_ gating property of Cx43/Cx43 gap junctional channels. Gap junctional channel: Cx43/Cx43. Internal and external solution: equal as in Figure 2. Patch clamps: *R*_s(L)_ = *R*_s(R)_ = 1 MΩ; Δ*V*_s_ = 0 mV for *t*/s ∉ [2, 10], Δ*V*_s_ = −130 to +130 mV for *t*/s ∈ [2, 10]. **(A)** Junctional currents induced by *V*_j_ pulses. **(B)** Graph of the steady-state (*V*_j_, *G*_j_) relationship, where the points are recorded at *t* = 10 s.

For *homotypic* connexin-base gap junctional channel (*h, h*_¶_), hold *V*_j_ = 0 mV, then ***z***Q***c***_*h*_ = ***z***Q***c***_*h*_¶__ ⇒ E(***c***_*h*_; ***c***_*h*_¶__) = 0 and E(***c***_*h*_¶__; ***c***_*h*_) = 0. In this state, for homotypic *h* and *h*_¶_ of the connexin family, $\sigma_{h}>{\bar{\sigma }}_{h}$, $\sigma_{h_{¶}}>{\bar{\sigma }}_{h_{¶}}$, ϕ_*h*_ ≈ 0 and ϕ_*h*_¶__ ≈ 0, the gap junctional channel remains open (σ_*h*_ is the detected ionic density of *h*). When *V*_j_ > 0 mV and gradually increased, calcium ions in cell L gathered toward the inner gate of hemichannel *h*, resulting in the increase of *g*_*h*_ (gate opening degree of *h*), while hemichannel *h*_¶_ was the opposite, and finally led to the decline of the permeability of gap junctional channel (*h, h*_¶_) (calculated by Equation 10). When both sides are symmetric, it can be derived that the *G*_j_ is maximal at *V*_j_ = 0 mV and it decreases more or less symmetrically at increasing |*V*_j_| to lower nonzero conductance values (termed *residual conductance*). See Figures 4, 5, **7** for numerical simulation results, which are consistent with the electrophysiological experiment records (Barrio et al., 1992; Quan et al., 2010).

**Figure 6:**
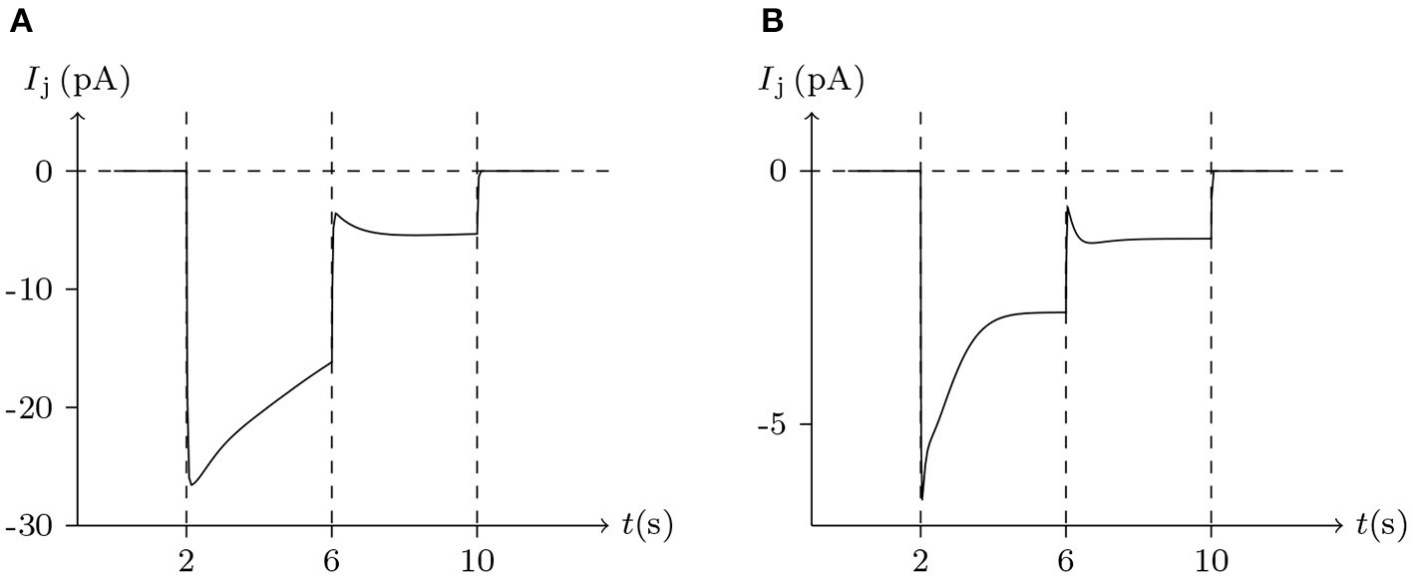
Numerical simulation of the comparison between Cx32/Cx32 and Cx43/Cx43 gap junctional channels. Patch clamps: *R*_s_ = 0.3 MΩ; Δ*V*_s_ = 0 mV for *t*/s ∉ [2, 10], Δ*V*_s_ = 100 mV for *t*/s ∈ [2, 6], Δ*V*_s_ = 20 mV for *t*/s ∈ [6, 10]. **(A)** Gap junctional channel: Cx32/Cx32. External and internal solution: equal as in Figure 4. **(B)** Gap junctional channel: Cx43/Cx43. External and internal solution: equal as in Figure 5.

Considering human Cx32/Cx32 (simulated in Figure 4) and rat Cx43/Cx43 junctions as examples (simulated in Figure 5), Cx43/Cx43 junctional conductance changes more rapidly than Cx32/Cx32 at the same *V*_j_ (Revilla et al., 1999) (simulated in Figure 6).

The above simulations support that the gap junction model in this paper applies to homotypic junctions. Next, we examined whether the gap junction model could predict the properties of heteromeric junctions based on the parameters of homotypic junctions. *Heterotypic* gap junctions demonstrated asymmetries in voltage sensitivities (Quan et al., 2010), as the *G*_j_/*V*_j_ currents of heteromeric gap junctions are generally asymmetric of *V*_j_ = 0.

Immunocytochemical and immunoblot analyses of retinal pigment epithelial cells identified Cx43 and Cx46 as the connexins mediating gap junctional intercellular communication. Homotypic (Cx43/Cx43 or Cx46/Cx46) or heterotypic (Cx43/Cx46) gap junctions may exist. Here, we simulated the response kinetics of gap junctions formed by pairing cells expressing Cx43 and Cx46 individually.

**Figure 7:**
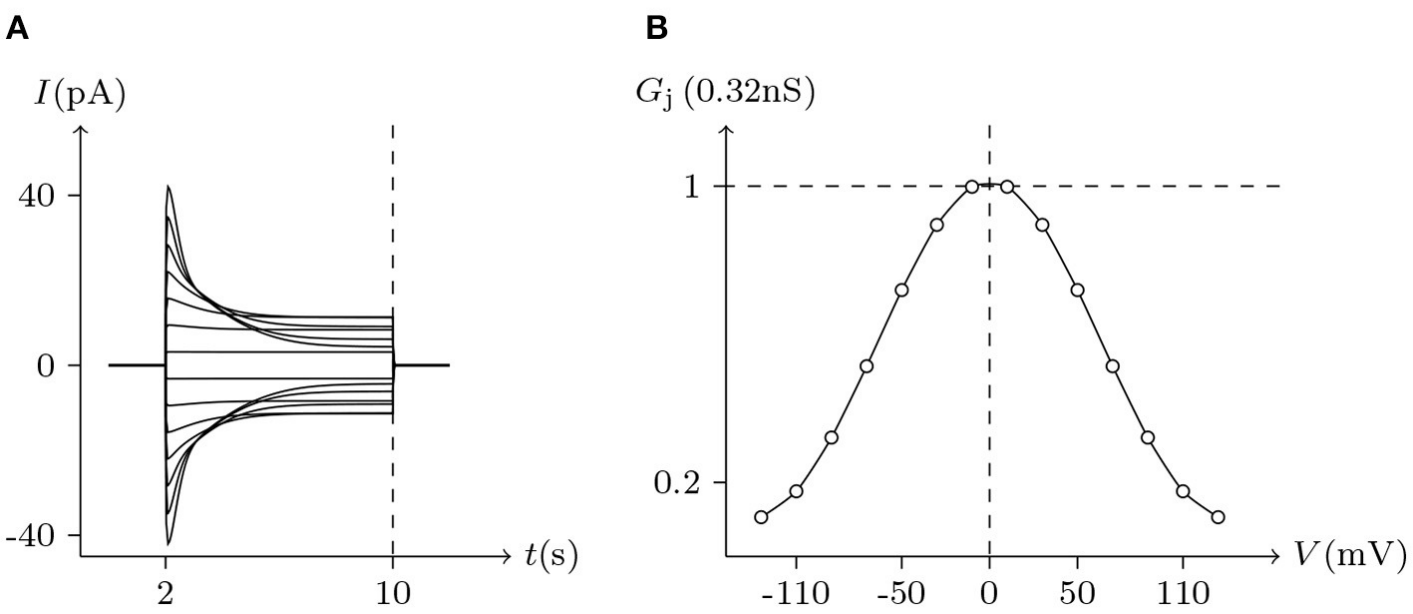
Simulated *V*_j_ gating property of Cx46/Cx46 gap junctional channels. Gap junctional channel: Cx46/Cx46. Internal and external solution: equal as in Figure 2. Patch clamps: *R*_s(L)_ = *R*_s(R)_ = 0.3 MΩ; Δ*V*_s_ = 0 mV for *t*/s ∉ [2, 10], Δ*V*_s_ = −130 to +130 mV for *t*/s ∈ [2, 10]. **(A)** Junctional currents induced by *V*_j_ pulses. **(B)** Graph of the steady-state (*V*_j_, *G*_j_) relationship, where the points are recorded at *t* = 10 s.

In previous reports, no significant hemichannel currents were observed in cells expressing Cx43. In contrast, large outward hemichannel currents were observed in cells expressing Cx46 (simulated in Figure 2). Both Cx43 and Cx46 readily form homotypic gap junctions when expressed in Xenopus oocytes. The junctional conductances of Cx43/Cx43 and Cx46/Cx46 were similar and generally symmetric of *V*_j_ = 0 mV. Conversely, the junctional conductances at *V*_j_ = −120 mV revealed significant differences between these two connexin-based junctions (Quan et al., 2010). This phenomenon is very consistent with the results of our model simulation (see Figures 5, 7).

**Figure 8:**
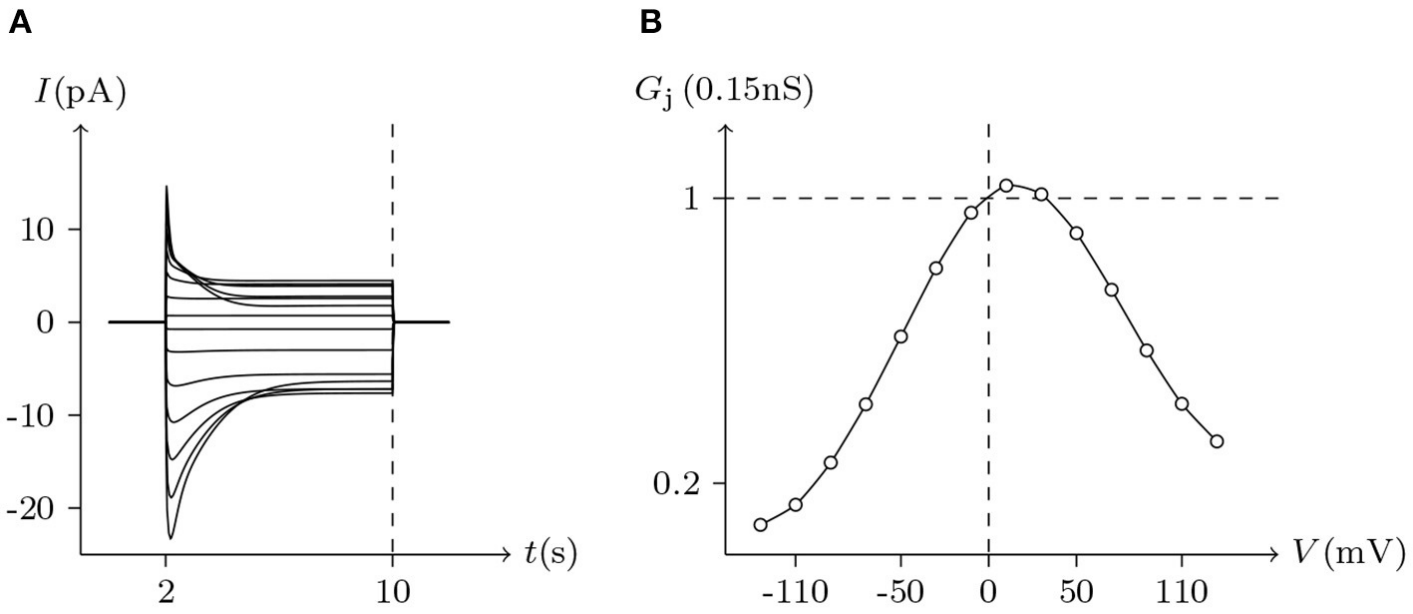
Simulated *V*_j_ gating property of Cx43/Cx46 heterotypic junctional channels. Gap junctional channel: Cx43/Cx46 (Cx43 expressed in cell L, Cx46 expressed in cell R). Internal and external solution: equal as in Figure 2. Patch clamps: *R*_s(L)_ = *R*_s(R)_ = 1.6 MΩ; Δ*V*_s_ = 0 mV for *t*/s ∉ [2, 10], Δ*V*_s_ = −130 to +130 mV for *t*/s ∈ [2, 10]. **(A)** Junctional currents induced by *V*_j_ pulses. **(B)** Graph of the steady-state (*V*_j_, *G*_j_) relationship, in which the points are recorded at *t* = 10 s.

On the other hand, Cx43 and Cx46 can also form heterotypic gap junctions. *G*_j_ of heterotypic Cx43/Cx46 junctions were smaller than homotypic Cx46/Cx46 junctions, suggesting that the building efficiency of heterotypic channels was lower. Furthermore, heterotypic Cx43/Cx46 junctions displayed a much larger gating asymmetry than was predicted from Cx43/Cx43 and Cx46/Cx46 junctions (Quan et al., 2010). However, based on our model of gap junctional channels, this gating asymmetry was indeed predictable. Substituting the parameters of Cx43/Cx43 and Cx46/Cx46 junctions into the model, the simulation results of Cx43/Cx46 junctions (see Figure 8) are almost consistent with physiological experiments.

#### 4.3.2. Formed by Innexins

In neural systems, different variants of the innexin Shaking B (ShakB), expressed in adjacent cells, can form heterotypic gap junctions as rectifying electrical synapses. In *Drosophila*, rectifying electrical synapses are formed by ShakB neural+16 (ShakBN16) and ShakB Lethal (ShakBL). ShakBN16 is expressed presynaptically and ShakBL is expressed postsynaptically (Phelan et al., 2009).

**Figure 9:**
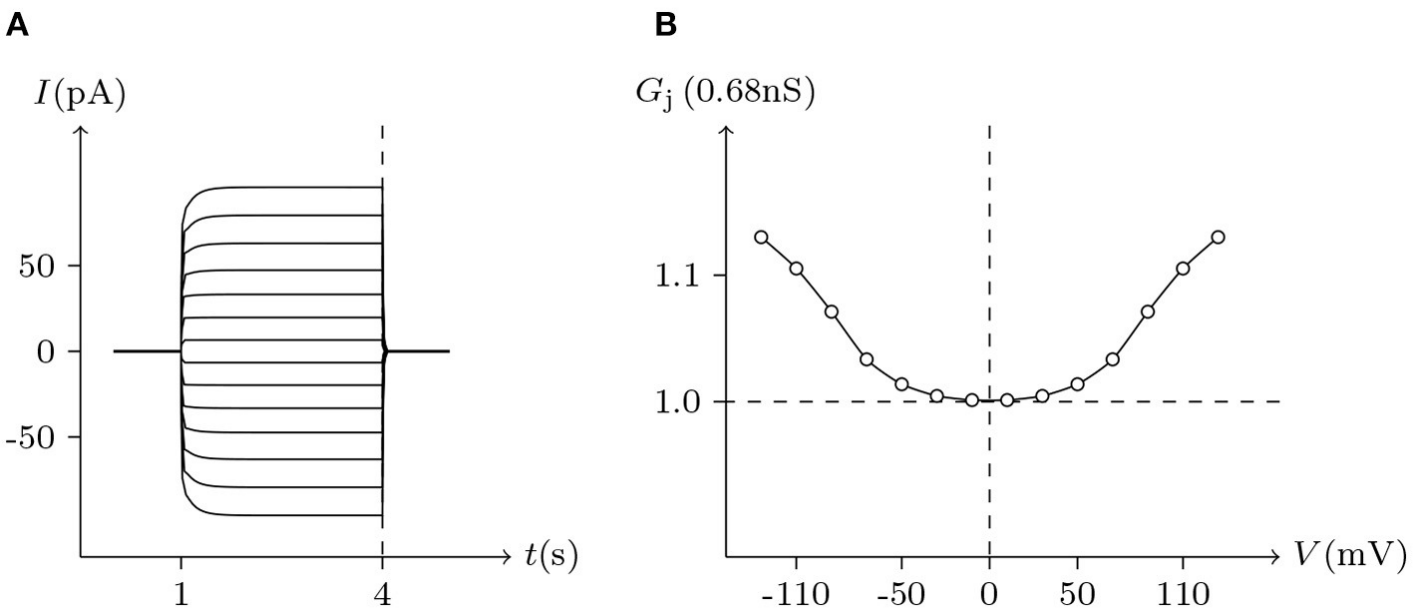
Simulated *V*_j_ gating property of ShakBL/ShakBL junctional channels. Gap junctional channel: ShakBL/ShakBL. Internal solution: I-2. External solution: Barth's. Patch clamps: *R*_s(L)_ = *R*_s(R)_ = 0.3 MΩ; Δ*V*_s_ = 0 mV for *t*/s ∉ [1, 4], Δ*V*_s_ = −130 to +130 mV for *t*/s ∈ [1, 4]. **(A)** Junctional currents induced by *V*_j_ pulses. **(B)** Graph of the steady-state (*V*_j_, *G*_j_) relationship, in which the points are recorded at *t* = 4 s.

**Figure 10:**
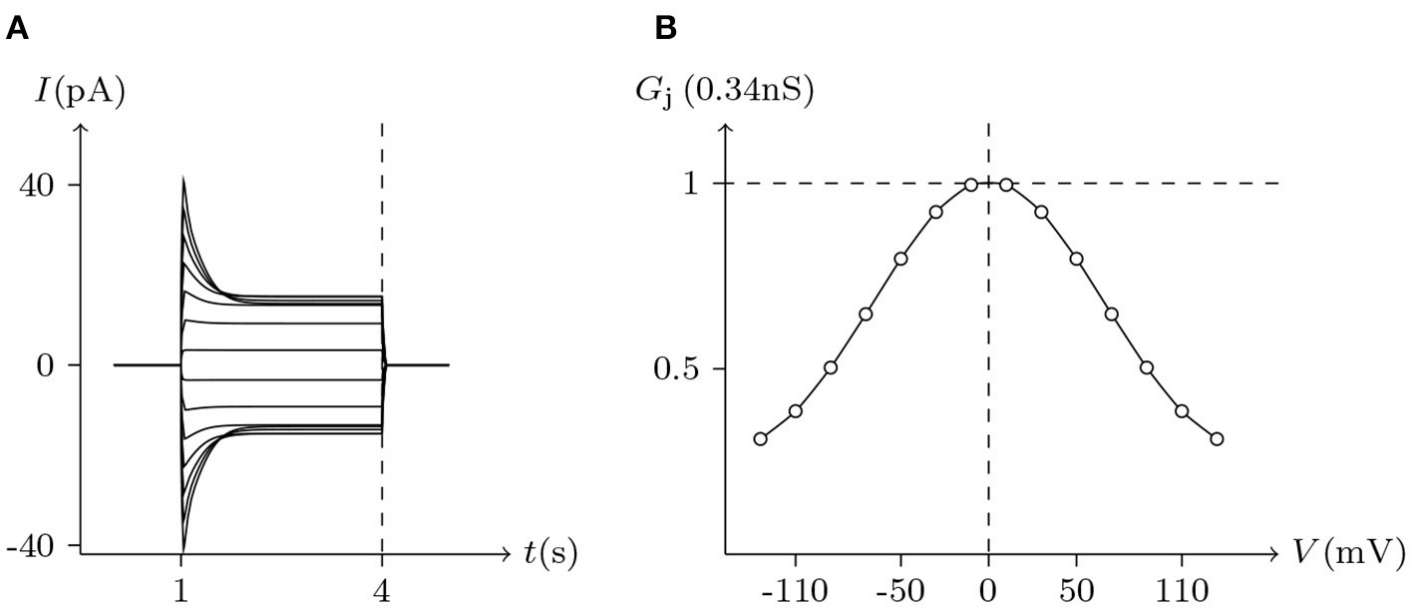
Simulated *V*_j_ gating property of ShakBN16/ShakBN16 junctional channels. Gap junctional channel: ShakBN16/ShakBN16. Internal solution: I-2. External solution: Barth's. Patch clamps: *R*_s(L)_ = *R*_s(R)_ = 0.3 MΩ; Δ*V*_s_ = 0 mV for *t*/s ∉ [1, 4], Δ*V*_s_ = −130 to +130 mV for *t*/s ∈ [1, 4]. **(A)** Junctional currents induced by *V*_j_ pulses. **(B)** Graph of the steady-state (*V*_j_, *G*_j_) relationship, in which the points are recorded at *t* = 4 s.

The voltage sensitivity of ShakBN16/ShakBN16 homotypic junctional channels (simulated in Figure 9) is not significant (Marks, 2012). As *V*_j_ increases, steady-state *G*_j_ slightly rises. In contrast, steady-state *G*_j_ of ShakBL/ShakBL homotypic junctional channels (simulated in Figure 10) declines with increasing |*V*_j_| (Phelan et al., 2009).

**Figure 11:**
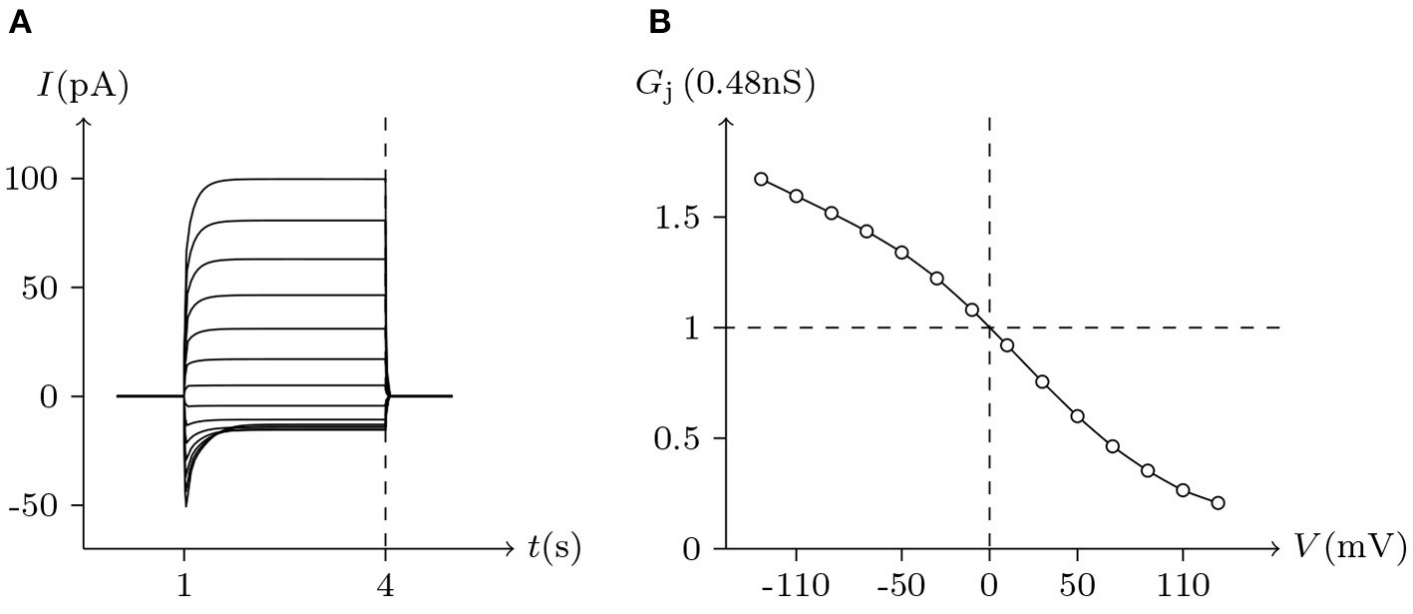
Simulated *V*_j_ gating property of ShakBL/ShakBN16 junctional channels. Gap junctional channel: ShakBL/ShakBN16 (ShakBL expressed in cell L, ShakBN16 expressed in cell R). Internal solution: I-2. External solution: Barth's. Patch clamps: *R*_s(L)_ = *R*_s(R)_ = 0.3 MΩ; Δ*V*_s_ = 0 mV for *t*/s ∉ [1, 4], Δ*V*_s_ = −130 to +130 mV for *t*/s ∈ [1, 4]. **(A)** Junctional currents induced by *V*_j_ pulses. **(B)** Graph of the steady-state (*V*_j_, *G*_j_) relationship, where the points are recorded at *t* = 4 s.

ShakBL/ShakBN16 heterotypic junctional channels are asymmetrically gated in response to transjunctional voltage, which differs significantly from ShakBL/ShakBL and ShakBN16/ShakBN16 homotypic junctions Marks (2012). Depolarizing *V*_s_ steps applied to the ShakBN16-expressing cells induced a large junctional current *I*_j_. By contrast, when ShakBL-expressing cells are subjected to depolarizing *V*_s_, the induced current is of low magnitude. Figure 11 simulates the relationship between *G*_j_ and *V*_j_ for ShakBL/ShakBN16 heterotypic channels. Steady-state *G*_j_ increases in a sigmoidal fashion as the cells expressing ShakBN16 were depolarized or the ShakBL-expressing cells were hyperpolarized relative to their heterotypic partners.

**Figure 12:**
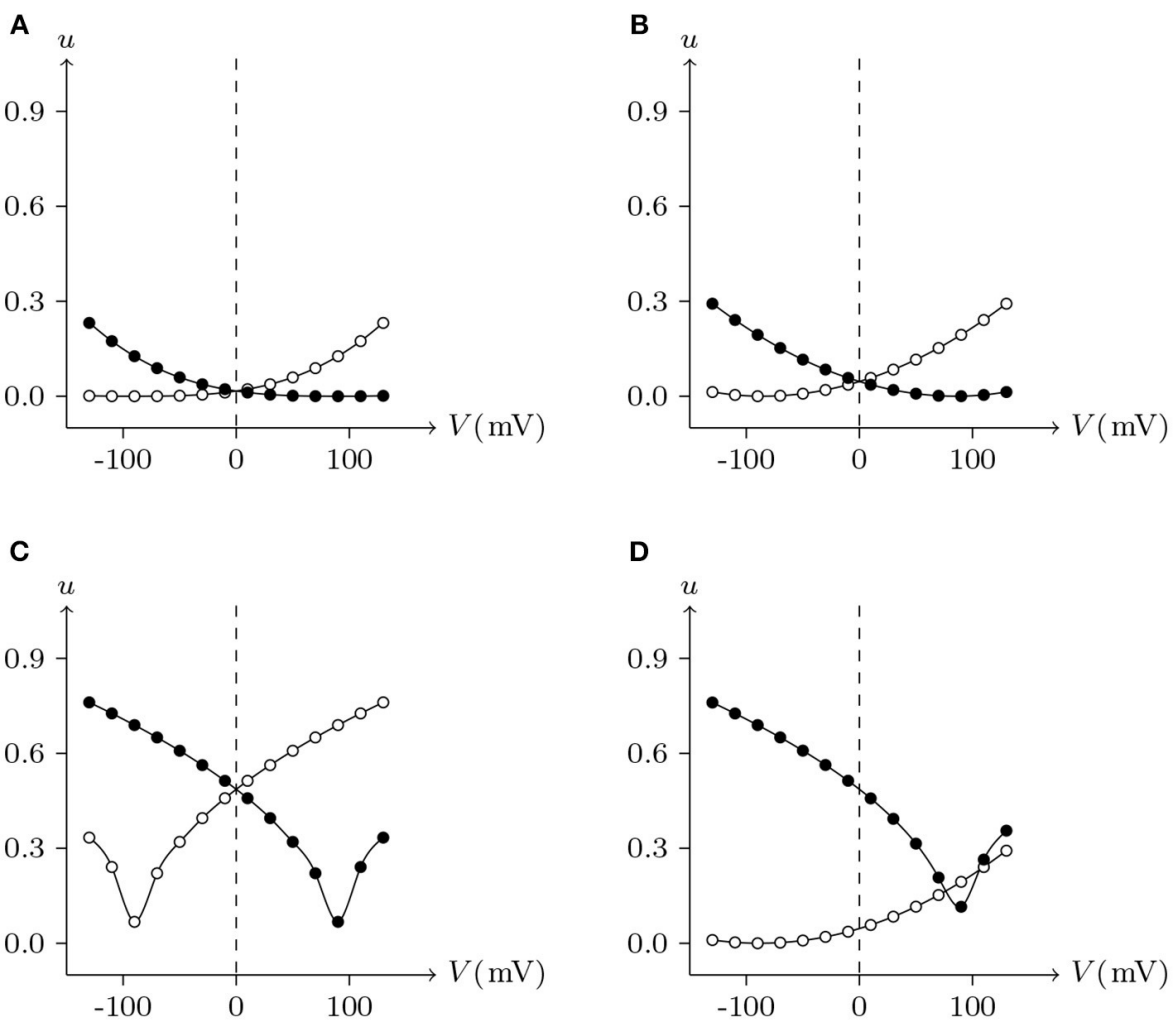
Interactions of docking hemichannels. The points are the values of the steady-state (*V*_j_, *u*_*h*_) and (*V*_j_, *u*_*h*_¶__) recorded in junctional channels (*h, h*_¶_), white for *h*, black for *h*_¶_. All parameters are the same as above. **(A)** Cx32/Cx32, **(B)** ShakBL/ShakBL, **(C)** ShakBN16/ShakBN16, **(D)** ShakBL/ShakBN16.

For junctional channel (*h, h*_¶_), if both *u*_*h*_ and *u*_*h*_¶__ are close to 0, as *h* and *h*_¶_ are relatively independent, then the voltage sensitivity of (*h, h*_¶_) is significant, such as ShakBL/ShakBL (see Figures 9, 12B), and other connexin gap junctional channels in this paper (e.g., Cx32/Cx32, see Figures 4, 12A). If both *u*_*h*_ and *u*_*h*_¶__ are large, as the interaction between *h* and *h*_¶_ is significant, the opening of one will prevent the closing of the other, and the closing of the one will also prevent the opening of the other. Particularly, when the two are in equilibrium, the junctional channel shows no voltage sensitivity. If *u*_*h*_ is much greater than *u*_*h*_¶__, as the effect of *h* on *h*_¶_ is much greater than that of *h*_¶_ on *h*, then the change of junctional channel permeability mainly depends on *h*, and vice versa. This explains why: the voltage sensitivity of ShakBN16/ShakBN16 homotypic junctional channels is not significant near *V*_j_ = 0 mV, and as |*V*_j_| increases, steady-state *G*_j_ slightly rises (see Figure 12C). ShakBL/ShakBN16 is special, where almost *u*_ShakBN16_ > *u*_ShakBL_ except in the vicinity of *V*_j_ = 90 mV, and *u*_ShakBN16_ ≫ *u*_ShakBL_ for *V*_j_ < 0 mV (see Figure 12D). So, in ShakBL/ShakBN16, ShakBN16 is dominant, especially in the range of *V*_j_ < 0 mV. Thus, the $\left(V_{\text{j}},\bar{G_{\text{j}}}\right)$ curve of ShakBL/ShakBN16 is inverse sigmoidal, as described above.

As observed, the nature of Innexin ShakB differs greatly from that of the connexin family. However, their physiological phenomena can be successfully simulated with the gap junctional channel model. Moreover, the parameters are unified without contradiction.

#### 4.3.3. Formed by Pannexins

The properties of non-junctional Oocytes Px1 simulated and analyzed above will not be repeated here.

Pannexins can form intercellular channels in paired oocytes. Pannexin 1 (Px1) alone and in combination with pannexin 2 (Px2) can form intercellular channels. Px1/Px1 pairs display a remarkable insensitivity to transjunctional voltage. At higher transjunctional voltage (|*V*_j_/mV| ≫ 0), the conductance of pannexin intercellular channels displays a slight but brief reduction (Bruzzone et al., 2003).

**Figure 13:**
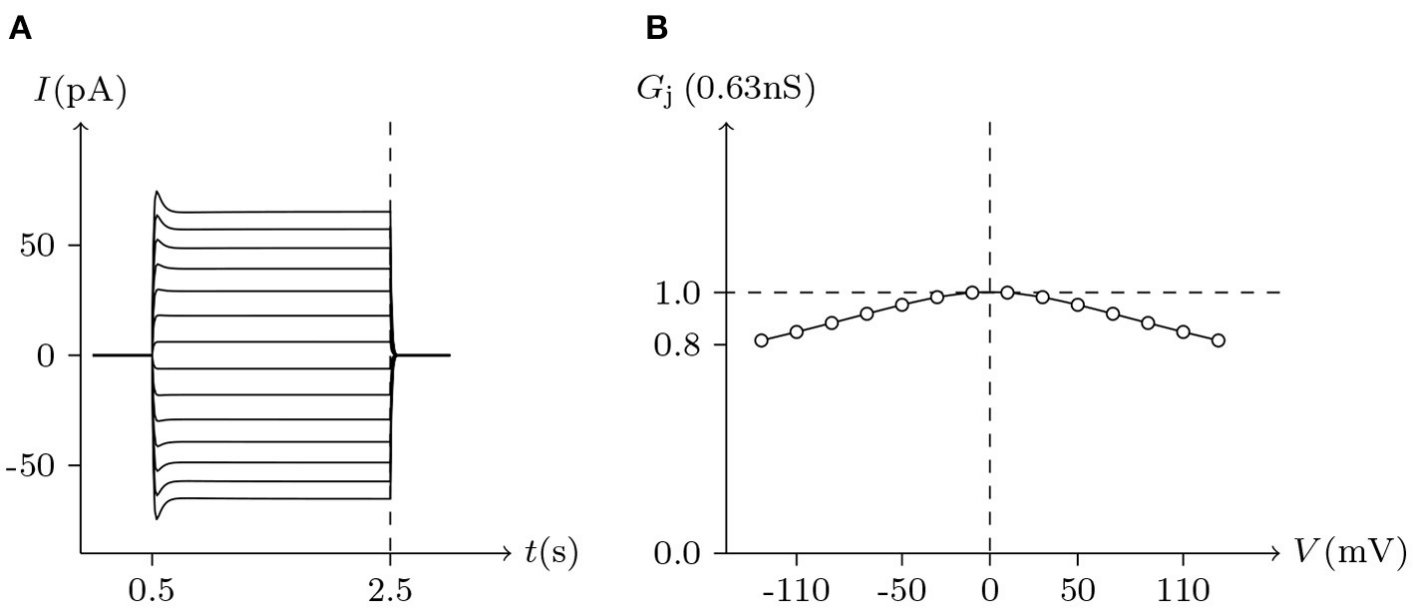
Simulation of *V*_j_ gating property of Px1/Px1 junctional channels. Gap junctional channel: Px1/Px1. Internal and external solution: equal as in Figure 3. Patch clamps: *R*_s(L)_ = *R*_s(R)_ = 0.3 MΩ; Δ*V*_s_ = 0 mV for *t*/s ∉ [0.5, 2.5], Δ*V*_s_ = −130 to +130 mV for *t*/s ∈ [0.5, 2.5]. **(A)** Junctional currents induced by *V*_j_ pulses. **(B)** Graph of the steady-state (*V*_j_, *G*_j_) relationship, where the points are recorded at *t* = 2.5 s.

These phenomena simulated in Figure 13, further demonstrate the applicability of the gap junction model.

**Figure 14:**
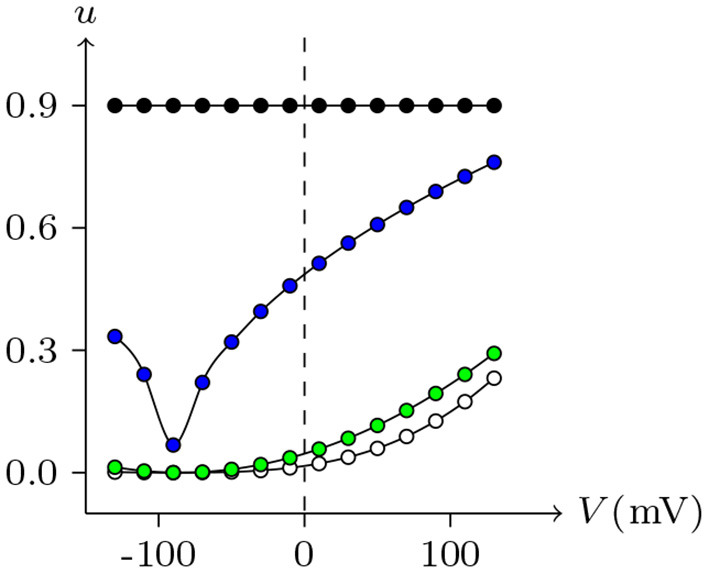
Interactions of symmetrical docking hemichannels. Because of symmetry, only one side of the data are shown here. The points are the values of the steady-state (*V*_j_, *u*_*h*_) recorded in junctional channels, white for Cx32/Cx32, green for ShakBK/ShakBL, blue for ShakBN16/ShakBN16, black for Px1/Px1. All parameters are the same as above.

By observing the interaction of the paired hemichannels (Px1_L_, Px1_R_) which form Px1/Px1 junctional channels, it can be seen that for any *V*_j_, there is *u*_Px_1__L__ = *u*_Px_1__R__ = 0.9. The hemichannels on both sides almost completely lose their sensitivity, which is quite different from most hemichannels of connexin and innexin families (see Figure 14 for Cx32, ShakBL, ShakBN16, and Px1), and it is also the reason why Px1/Px1 pairs appear to be almost insensitive to *V*_j_.

#### 4.3.4. Permeability

Ion substitution is generally used to study the selective permeability of channels. Scholars often choose to substitute anions significantly different in size or aqueous mobility, such as glutamate, which has only one-fifth the aqueous mobility of chloride. Results of physiological experiments indicated that, although Cx32 channels mainly transport cations, they are also permeable to Cl^−^ (Suchyna et al., 1999).

**Figure 15:**
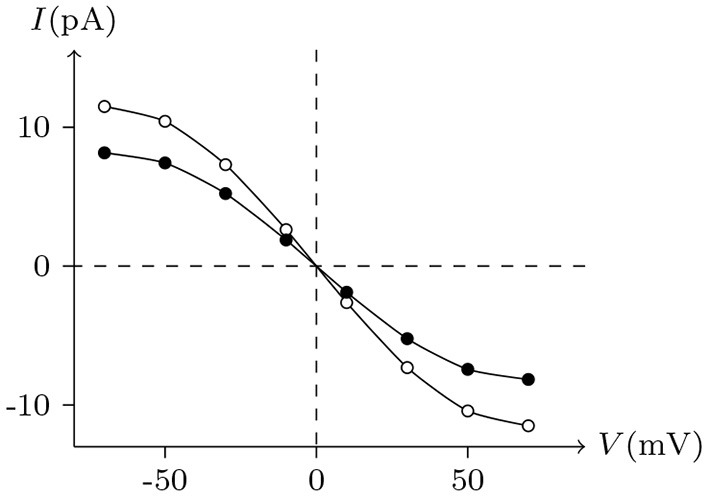
Numerical simulation of the experiments to study the permeability of Cx32/Cx32 junctions to Cl^−^. White points (junctional currents and voltages) are recorded at *t* = 20 s in the experiment of Figure 4. Replacing KCl with K-glu, while keeping the other parameters constant, allows obtaining the black points.

Ion permeabilities of hemichannels and gap junctional channels can be calculated (see Table 2) by fitting the permeability curves of our model (see Figure 15). Essentially, the permeability of junctional channel (*h, h*_¶_) depends on the permeabilities of hemichannels *h* and *h*_¶_.

#### 4.3.5. Signal Transmission

The docking between hemichannels forms gap junctions that allow the passage of ions, generating communication.

However, there is no model to describe how variations in hemichannel assembly can generate the variety of communication properties of gap junctions.

**Figure 16:**
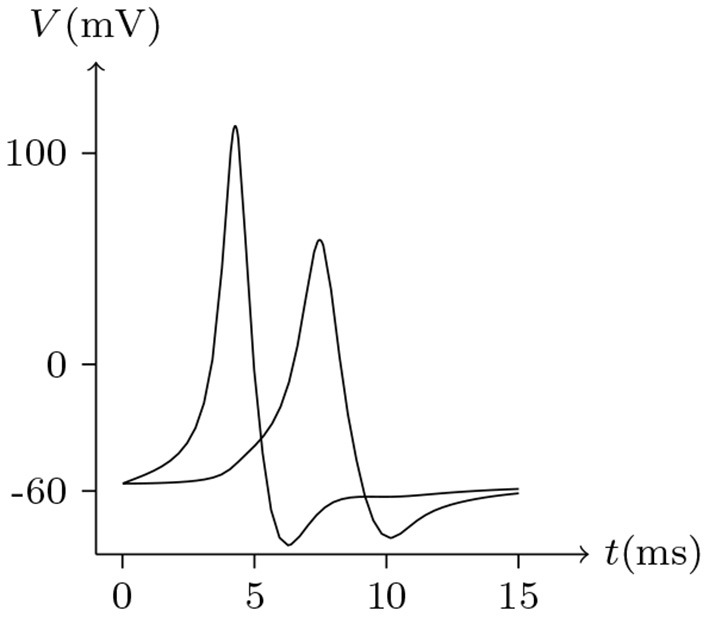
Numerical simulation of time delay and attenuation of signal transmission via Cx36/Cx36 junction. Gap junction: Cx36/Cx36. Ion channel list of cell L and R: Na_v_, K_v_, Cl_v_. Internal solution: I-4. External solution: E-1. Patch clamp impaling cell L: ${ı}_{\text{s}}=0.05\text{A}/{\text{m}}^{2}$ for *t*/ms ∈ [0, 5], ${ı}_{\text{s}}=0\text{A}/{\text{m}}^{2}$ for *t*/ms ∉ [0, 5].

**Figure 17:**
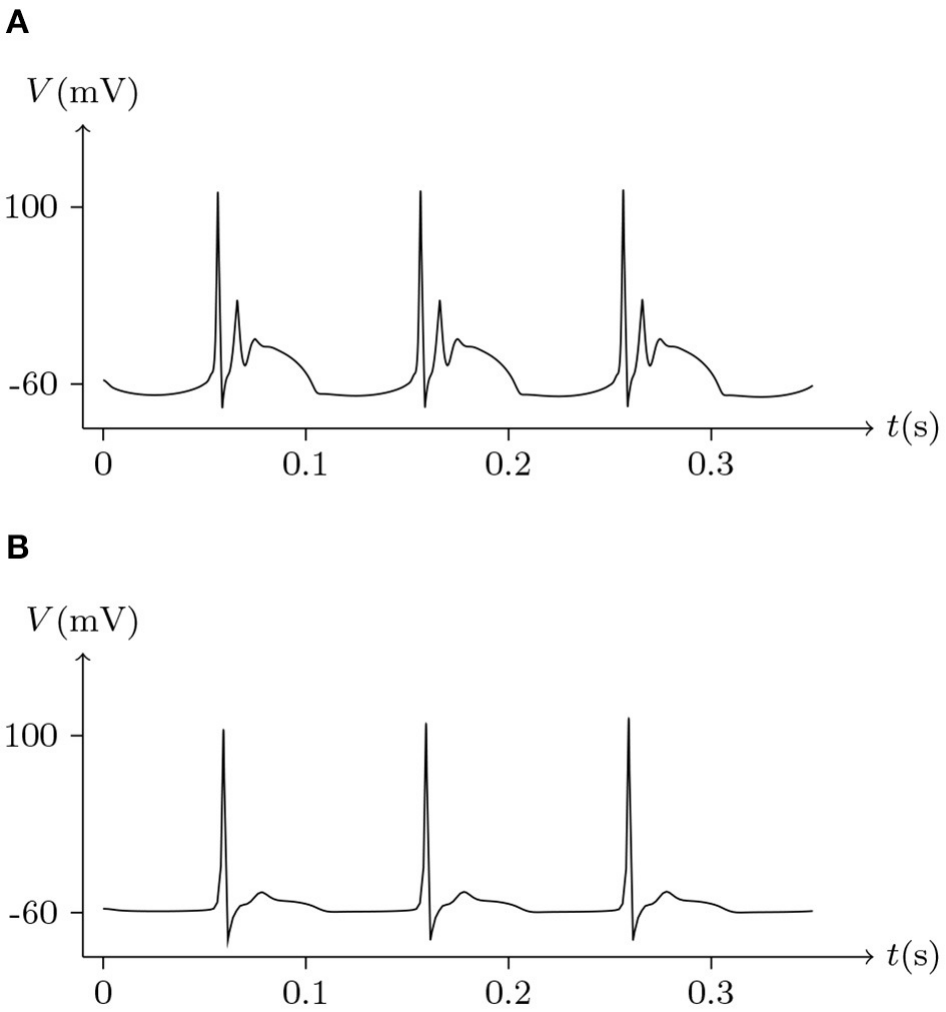
Numerical simulation of bursting signal transmission via Cx36/Cx36 junction. Patch clamp impaling cell L: ${ı}_{\text{s}}=-0.2\text{A}/{\text{m}}^{2}\cdot \sin20\pi t/\text{s}$. Gap junction: Cx36/Cx36. Ion channel list of cell L and R: Na_v_, K_v_, Cl_v_. Internal solution: I-4. External solution: E-1. **(A)** Membrane potential of cell L. **(B)** Membrane potential of cell R.

Electrical synapses are often considered symmetrical structures, with presynaptic and postsynaptic sites considered as mirror images of each other. In mammals, Cx36 is widely expressed in neurons (Steyn-Ross et al., 2012), which only forms homotypic intercellular channels (Teubner et al., 2000). Figures 16, 17 simulate signal transmission by Cx36/Cx36 junction.

**Figure 18:**
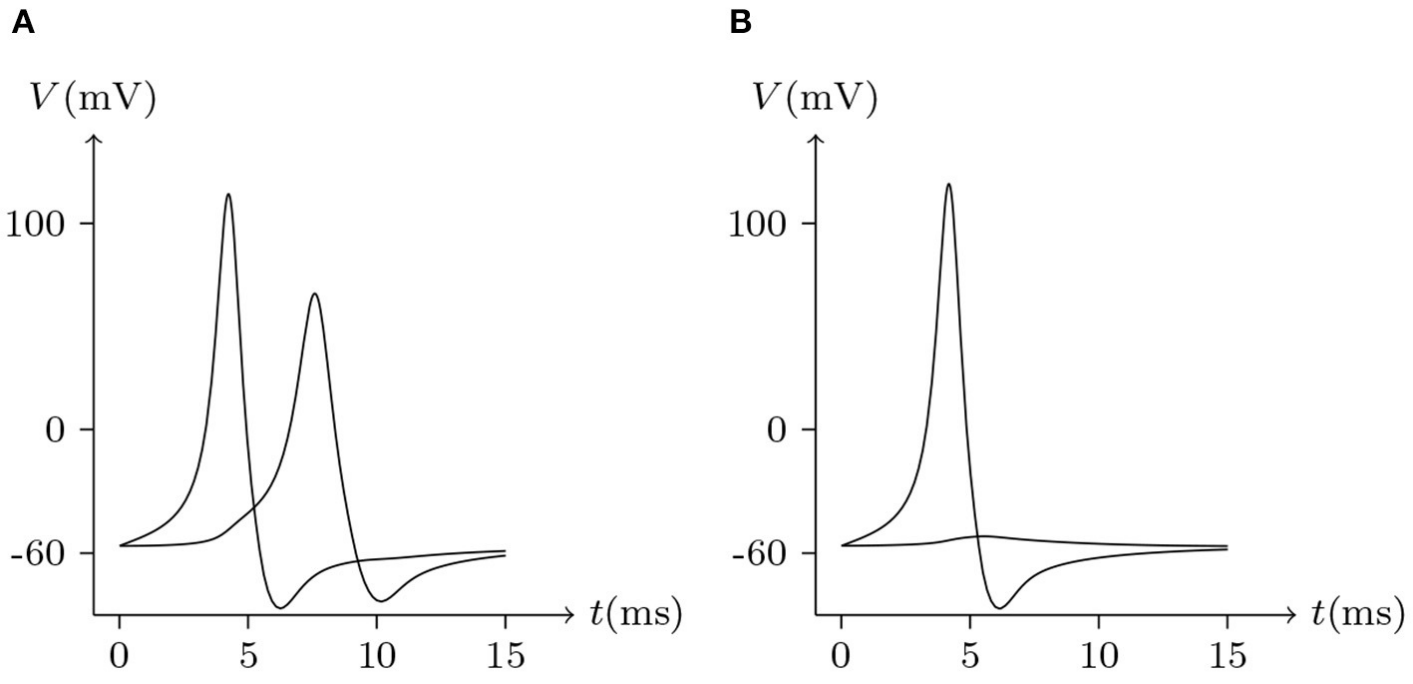
Numerical simulation signal transmission via Cx43/Cx46 junction. Gap junction: Cx43/Cx46. Ion channel list of cell L and R: Na_v_, K_v_, Cl_v_. Internal solution: I-4. External solution: E-1. **(A)** Patch clamp impaling cell L (expressing Cx43): ${ı}_{\text{s}}=0.05\text{A}/{\text{m}}^{2}$ for *t*/ms ∈ [0, 5], ${ı}_{\text{s}}=0\text{A}/{\text{m}}^{2}$ for *t*/ms ∉ [0, 5]. **(B)** Patch clamp impaling cell R (expressing Cx46): ${ı}_{\text{s}}=0.05\text{A}/{\text{m}}^{2}$ for *t*/ms ∈ [0, 5], ${ı}_{\text{s}}=0\text{A}/{\text{m}}^{2}$ for *t*/ms ∉ [0, 5].

It has been reported that Cx35 and Cx34.7 can form heterotypic junctions, with Cx35 expressed presynaptically and Cx34.7 expressed postsynaptically (Rash et al., 2013). Asymmetry in the molecular composition of adjoining connexons allows electrical rectification at some gap junctions (Phelan et al., 2009). Thus, molecular asymmetries in gap junctions underpin the complexity of their functional properties. and suggest that the signal transmission of electrical synapses is not necessarily symmetrical. This difference has been observed in simultaneous recordings in physiological experiments (Rash et al., 2013). As mentioned above, connexins mediating gap junctional intercellular communication in retinal pigment epithelial cells have been identified as Cx43 and Cx46. With the parameters employed, we went onto simulate the non-mirrored signal transmission of Cx43/Cx46 junctions, as illustrated in Figure 18. Also, the delay of electrical signal transmission in numerical experiments of heterotypic junctions is evident, which can be observed in electrophysiological experiments (Giaume and Korn, 1984).

## 5. Conclusion

Electrical synaptic transmission is considered an essential form of interneuronal communication, which is mediated by gap junctions. This method of communication is considered a form of direct transmission. Gap junctions transfer ions and other small molecules but not electrons, like the plasmodesmata of plants and septal pores of fungi. Electrical signal transmission is the result of ion transmission. Thus, a rational model should be able to describe and simulate the ion flow via gap junctions. Furthermore, each gap junctional channel is formed by the docking of hemichannels, which can be voltage gated by depolarization in the non-junctional plasma membrane. Moreover, most unpaired hemichannels are functional.

However, a proper model for these functionalities is lacking. And no models have yet been able to correlate the two distinct gating mechanisms of hemichannels and junctional channels.

This study verified the applicability of the passive transmembrane ion transport model to hemichannels in non-junctional plasma membranes after simulating the typical electrophysiological phenomena of hemichannels, such as activation and ion blockage of unpair hemichannels. Moreover, by model-fitting studies, we can obtain detailed parameters, some of which cannot directly be measured in electrophysiological experiments, but can reveal several properties of unpaired hemichannels. For example, the sensitivity parameters indicate that Cx32 hemichannels are sensitive to intracellular Ca^2+^, while being even more sensitive to intracellular Mg^2+^. Another example is the filter parameters that indicate the function of unpaired Px1 channels on ion transport is different from that of Cxs and Ixs, which is reflected in the change in ion selectivity after activation.

On this basis, we have established a gap junctional channel model, which describes the docking of hemichannels, and simulates the homotypic and heterotypic gap junctions formed by connexins, innexins, and pannexins. Comparing the simulation results with the actual measurement data, we believe that this model is widely applicable for multiple types of gap junctions, even though the molecular structures and electrophysiological properties of Cxs, Ixs, and Pxs differ. It also indicates that the gating mechanism of junctional channels is completely determined by docking hemichannels and can be calculated. Using this model, electrical signal transmission can be simulated successfully, where signal attenuation and delay can be observed. In particular, the non-mirrored signal transmission can be observed in numerical experiments of heterotypic junctions. These results that are consistent with the phenomenon of electrophysiological experiments, indicate that the proposed model is widely applicable to different types of gap junctions.

In summary, our work unified the gating mechanisms of hemichannels and gap junctional channels which were previously considered unrelated, and explained their associated electrophysiological properties based on ion passivity transport. Some properties that cannot be directly observed by electrophysiological experiments can be reasonably inferred by our model, which may provide clues for us to further explore the physiological functions of hemichannels and junctional channels.

## Data Availability Statement

The original contributions presented in the study are included in the article, further inquiries can be directed to the corresponding author.

## Author Contributions

QW: conceptualization, software, and writing. SL: supervision. All authors contributed to the article and approved the submitted version.

## Conflict of Interest

The authors declare that the research was conducted in the absence of any commercial or financial relationships that could be construed as a potential conflict of interest.
